# *Salmonella* Extracellular Polymeric Substances Modulate Innate Phagocyte Activity and Enhance Tolerance of Biofilm-Associated Bacteria to Oxidative Stress

**DOI:** 10.3390/microorganisms8020253

**Published:** 2020-02-13

**Authors:** Mark M. Hahn, John S. Gunn

**Affiliations:** 1 Center for Microbial Pathogenesis, Abigail Wexner Research Institute at Nationwide Children’s Hospital, Columbus, OH 43205, USA; mark.hahn@nationwidechildrens.org; 2 Infectious Diseases Institute, The Ohio State University, Columbus, OH 43210, USA; 3 Department of Pediatrics, College of Medicine, The Ohio State University, Columbus, OH 43210, USA

**Keywords:** *Salmonella*, biofilm, innate immunity, extracellular polymeric substances

## Abstract

*Salmonella enterica* serovar Typhi causes 14.3 million acute cases of typhoid fever that are responsible for 136,000 deaths each year. Chronic infections occur in 3%–5% of those infected and *S.* Typhi persists primarily in the gallbladder by forming biofilms on cholesterol gallstones, but how these bacterial communities evade host immunity is not known. *Salmonella* biofilms produce several extracellular polymeric substances (EPSs) during chronic infection, which are hypothesized to prevent pathogen clearance either by protecting biofilm-associated bacteria from direct humoral attack or by modulating innate phagocyte interaction with biofilms. Using wild-type and EPS-deficient planktonic and biofilm *Salmonella*, the direct attack hypothesis was tested by challenging biofilms with human serum and antimicrobial peptides. Biofilms were found to be tolerant to these molecules, but these phenotypes were independent of the tested EPSs. By examining macrophage and neutrophil responses, new roles for biofilm-associated capsular polysaccharides and slime polysaccharides were identified. The *S.* Typhi Vi antigen was found to modulate innate immunity by reducing macrophage nitric oxide production and neutrophil reactive oxygen species (ROS) production. The slime polysaccharides colanic acid and cellulose were found to be immune-stimulating and represent a key difference between non-typhoidal serovars and typhoidal serovars, which do not express colanic acid. Furthermore, biofilm tolerance to the exogenously-supplied ROS intermediates hydrogen peroxide (H_2_O_2_) and hypochlorite (ClO^−^) indicated an additional role of the capsular polysaccharides for both serovars in recalcitrance to H_2_O_2_ but not ClO^−^, providing new understanding of the stalemate that arises during chronic infections and offering new directions for mechanistic and clinical studies.

## 1. Introduction

*Salmonella enterica* subspecies *enterica* serovar Typhi (*S.* Typhi) is a chronic pathogen of the gallbladder, where it forms biofilms anchored to cholesterol gallstones and encased in self-produced extracellular polymeric substances (EPSs) [1,2,3,4,5]. The conditions and location of these recalcitrant infections is both perplexing and problematic. Bile is rich in bile acids and bile salts with extensive immune-stimulating, antimicrobial, and detergent-like properties that can disrupt bacterial membranes, halt proton gradients, and induce redox stress [6,7]. How and why *S.* Typhi establishes chronic infections in such a repressive environment is not well understood [7]. Furthermore, bile is an important environmental signal that causes opposite effects between non-typhoidal and typhoidal *Salmonella*, the most prominent being those involved in host cell invasion [6,7,8,9]. The chronic biofilm lifecycle of *S.* Typhi is also problematic as it presents unique challenges for diagnosing infections [10,11,12,13], providing efficacious treatment [5,14], and the eradication of endemic disease [15,16,17]. However, these issues must be addressed because chronic carriers are the only known reservoir of *S.* Typhi and eradication of these infections will be an essential step in preventing the 136,000 deaths caused by 14.3 million cases of acute typhoid fever each year [15].

*S.* Typhi is a human-restricted pathogen. An important aspect that sets *S.* Typhi apart from *Salmonella enterica* subspecies *enterica* serovar Typhimurium (*S.* Typhimurium) and other non-typhoidal serovars is rampant genomic decay with pseudogenes representing roughly 5% of its genome, as well as specific gene acquisitions [18,19,20]. These adaptions resulted in host specialization and the ability to cause systemic disease, which begins primarily by the infection of M cells in the distal ileum. Subsequent invasion and persistence inside macrophages of Peyer’s Patches allows *S.* Typhi to disseminate to deep tissues, such as the liver [21,22]. From the liver, *S.* Typhi descends the hepatobiliary duct to the gallbladder and establishes acute or chronic infection [1,4]. Throughout this process, planktonic *S.* Typhi exhibit anti-inflammatory properties to avoid immune detection [23,24,25,26,27,28,29,30]. However, the intracellular lifecycle is a major factor in immune evasion, and biofilms—existing extracellularly—must have additional mechanisms to elicit immune modulation.

Many chronic pathogens are known to inhibit immune cell functions with a well-established link between biofilms and cellular suppression [31,32,33,34,35]. Specific EPSs are often attributed to these abilities, as EPSs have been found to be fundamental to recalcitrance to host immunity and pharmaceutical approaches. One of the most prominent examples of this phenomenon comes from *Pseudomonas aeruginosa* biofilms, which commonly infect cystic fibrosis and burn wound patients. Alginate, the major exopolysaccharide of *P. aeruginosa* biofilms, scavenges hypochlorite and inhibits innate immunity at multiple processes, including complement activation, polymorphonuclear chemotaxis, and phagocytosis by neutrophils and macrophages [36]. *Salmonella* biofilms produce various EPSs in vitro, and biofilm formation during chronic infection has been directly observed in vivo [3,4]. The asymptomatic nature of chronic biofilm infections suggests that *S.* Typhi EPSs have a role in altering innate immune activities to avoid detection. Therefore, we hypothesized that one or more of the EPSs are crucial for biofilm tolerance and contribute to the chronic pathogenicity of *S.* Typhi biofilms by skewing innate immune function(s). Broadly, this prediction presented two functional categories: (a) EPSs that protect biofilm-associated bacteria from innate immune functions that are otherwise inhibitory to planktonic *Salmonella* or (b) EPSs that have immune-modulating function(s) and thus alter the host response to biofilms. We have previously defined the major EPSs of *Salmonella* biofilms (curli fimbriae, colanic acid, cellulose, extracellular DNA, O antigen capsule, and Vi antigen) and characterized the role of each component for biofilm development in vitro [37]. Although these EPSs (particularly curli fimbriae) account for 90% of the biofilm biomass [38], the contribution of each EPS to resisting innate immune functions has not been thoroughly studied.

To test our hypothesis, we compared the outcomes of planktonic and biofilm-associated *Salmonella* when challenged with soluble innate immune molecules (normal human serum and antimicrobial peptides [AMPs]) and tested neutrophils and macrophages for functional responses to planktonic and biofilm *Salmonella*. Furthermore, by comparing wild-type (WT) and EPS-deficient biofilms, we present new evidence that *Salmonella* biofilm tolerance to the host oxidative burst is dependent on the Vi antigen, the O antigen capsule, and colanic acid.

## 2. Materials and Methods

### 2.1. Bacterial Strains and Growth Conditions

The *Salmonella* strains used in this study were the parental WT strains or derivatives of *S.* Typhimurium ATCC 14028 (JSG210) and *S.* Typhi Ty2 (JSG698, JSG4383) (Table 1). The latter WT *S.* Typhi was substituted when appropriate to correct for a known *rpoS* mutation in this strain [39]. At times, clinical isolates were also tested (Table 2). All clinical *S*. Typhi isolates tested positive for Vi antigen by serum agglutination tests. The O antigen capsule mutant does not alter the LPS structure. All planktonic and biofilm cultures were grown in tryptic soy broth (TSB). Planktonic cells were collected from 16-hour overnight broth cultures. Biofilms were cultured in 96-well polypropylene microtiter plates coated with 500 µg of cholesterol. To initiate biofilm growth, overnight planktonic bacteria were normalized to OD_490_ = 0.65 in TSB and then diluted 1:6 in TSB and incubated at 37 °C for 3 hours in a static 12-well polypropylene plate. After 3 hours, static cultures were diluted 1:2500 in TSB and distributed (200 µL/well) to the aforementioned cholesterol-coated wells and transferred to 30 °C. Biofilms were cultured on a nutator at 30 °C for 96 hours. Supernatants were replaced with fresh TSB once every 24 hours. Prior to experimental use, mature biofilms were washed with phosphate-buffered saline (PBS) to remove unattached and planktonic bacteria.

### 2.2. Mutant Generation

The Vi antigen was eliminated from a *S.* Typhi clinical isolate (JSG3074) by λ-Red mutagenesis [42]. The primer designs are detailed in Table 3. The marked *ΔtviB* gene deletion was transformed into *S.* Typhi JSG3074 carrying the λ-Red recombinase (creating strain JSG4097), and the antibiotic resistance marker was subsequently removed using pCP20 [43]. The final mutant (JSG4123) was verified by PCR and analysis by gel electrophoresis.

### 2.3. Sensitivity and Tolerance to Human Serum

Sensitivity to normal human serum was determined using WT planktonic *Salmonella* spp. Overnight broth cultures were normalized to 1.0 × 10^7^ colony forming units per milliliter (CFUs/mL) in 30% Human AB serum pooled from healthy male donors (Mediatech Inc.; Manassas, VA, USA). Heat-inactivated serum (56 °C for 30 minutes) was included as the negative control. Cultures were incubated at 37 °C for 3 hours on a nutator and then quantified by dilution platting and colony forming unit (CFU) enumeration.

After washing 2× with PBS, biofilms were challenged with 30% normal human serum or heat-inactivated serum. Serum-exposed biofilms were incubated at 37 °C for 3 hours on a nutator and then washed with PBS to remove residual serum. Viable bacteria remaining in the biofilm were enumerated by serial dilution platting.

### 2.4. Sensitivity and Tolerance to Antimicrobial Peptides

WT planktonic *Salmonella* spp. normalized to approximately 2.0 × 10^6^ CFUs/mL in TSB were used to determine the minimum inhibitory concentration (MIC) of the AMPs polymyxin B sulfate (Gibco; Billings, MT, USA) and melittin (Sigma-Aldrich; St. Louis, MO, USA). Concentrations were assayed in two-fold serial dilution series, and growth at 37 °C was monitored by OD_600_ on a SpectraMax M3 plate reader. The MICs for each AMP were determined by the lowest concentration tested that prevented detectable growth. Mature biofilms were washed and exposed to each AMP at concentrations 10× that of the WT planktonic MIC. Biofilms were incubated with each AMP at 37 °C for 2 hours on a nutator before PBS washing to remove trace peptides and enumeration by mechanical collection and serial dilution platting.

### 2.5. Biofilm Aggregate Collection

Mature biofilms aggregates were used for all host–response experiments and subsequent pathway analysis investigation. Aggregates were mechanically collected by scraping microtiter plate biofilms and normalized by total protein quantification (Bradford method). The reported multiplicity of infection (MOI) values refer to the biofilm aggregate protein equivalent (MOI_eq_) to that of planktonic bacteria at the reported MOI. Normalized aggregates were analyzed for size and granularity characteristics by flow cytometry (assay optimization only) using a BD FACSCanto II flow cytometer (BD Biosciences; San Jose, CA, USA).

### 2.6. Macrophage Nitric Oxide Response to Salmonella

The THP-1 cell line was maintained in Roswell Park Memorial Institute (RPMI) media supplemented with 10% fetal bovine serum (FBS) and 2 mM of l-glutamine. Prior to infection, THP-1 cells were washed and normalized in equivalent media lacking phenol red. Then, the cell line was infected with planktonic or biofilm samples (MOI_eq_ = 100, 4.0 × 10^5^ THP-1 cells total), and infections were synchronized by centrifugation. Cultures were incubated for 3 hours at 37 °C, 5% CO_2_. Supernatant nitric oxide (NO) was measured each hour by the Griess diazotization reaction, and viable extracellular CFUs remaining in the supernatant were quantified by serial dilution platting.

### 2.7. Neutrophil Reactive Oxygen Species Response to Salmonella

PLB-985 cells [44,45] were differentiated to a neutrophil-like phenotype [46,47] by 6-day incubation in Advanced RPMI supplemented with 0.5% N, N-Dimethylformamide, 0.5% FBS, 1% Nutridoma-SP (Roche; Mannheim, Germany), 2 mM L-glutamine, and 1× penicillin/streptomycin. Media was replaced on day 3. Differentiated PLB-985 cells were infected with planktonic or biofilm samples (MOI_eq_ = 50, 6.0 × 10^6^ PLB-985 cells total) that had been opsonized in 20% normal human serum for 20 minutes prior to infection. Uninfected PLB-985 cells were stimulated with phorbol 12-myristate 13-acetate (PMA) (final concentration of 1.0 × 10^−4^ mg/mL). All samples were supplied with luminol (final concentration of 500 µM); then, infections were synchronized by centrifugation. Reactive oxygen species (ROS) production at 37 °C was monitored in triplicate by luminol-dependent chemiluminescence measured every 2 minutes for 1 hour using a SpectraMax M3 plate reader. Area under the curve (AUC) was calculated for each condition and normalized to the AUC calculated for PMA-stimulated PLB-985 cells. Parallel infections were conducted to determine total CFUs remaining at 80 minutes post-infection. Gentamicin was not added to the media, and the PLB-985 cells were lysed by 0.1% sodium dodecyl sulfate treatment prior to serial dilution platting; thus, the reported CFUs represent total bacteria remaining from input after 80 minutes.

### 2.8. Sensitivity and Tolerance to Oxidative Species

Overnight cultures were normalized to approximately 2.0 × 10^6^ CFUs/mL in TSB and incubated at 37 °C on a rolling drum. Planktonic sensitivity to hydrogen peroxide (H_2_O_2_) and hypochlorite (ClO^−^) was initially assayed using WT *S.* Typhimurium or *S.* Typhi exposed to 0 mM, 5 mM, or 10 mM H_2_O_2_ or 0 μg/mL, 250 μg/mL, or 1000 μg/mL ClO^−^. Viable cells remaining after 30- and 60-minute exposure were enumerated by dilution platting.

The MIC of each oxidative species against planktonic *Salmonella* was determined by 16-hour growth curves (OD_600_). Starting cultures contained approximately 2.0 × 10^6^ CFUs/mL in TSB with H_2_O_2_ or ClO^−^ supplied at final concentrations ranging from 10 mM to 0.3125 mM or 1000 μg/mL to 15 μg/mL (respectively) in two-fold serial dilutions. Microtiter plates were incubated at 37 °C for 16 hours and growth was monitored by OD_600_. Readings were recorded every 30 minutes using a SpectraMax M3 plate reader. The MICs for each oxidative species were determined by the lowest concentration that prevented detectable growth.

Biofilm aggregates were challenged with H_2_O_2_ or ClO^−^ supplied at 1×, 10×, 25×, of 50× the experimentally-determined planktonic cell MIC and incubated at 37 °C for 2 hours on an orbital shaker. Viable biofilm aggregates were disrupted and enumerated before and after exposure by serial dilution platting.

## 3. Results

### 3.1. Biofilm Tolerance to Innate Immunity

#### 3.1.1. Each of the Four Major *Salmonella* Biofilm EPSs Contribute to Tolerance to Innate Immunity

**Figure 1:**
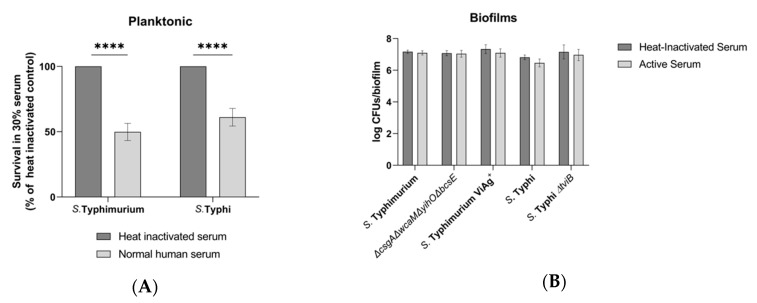
Sensitivity and tolerance to human serum. JSG698 was used for wild-type (WT) *S.* Typhi. (**A**) Growth from input of WT planktonic *Salmonella* following challenge with 30% normal human serum or heat-inactivated serum determined by colony forming unit (CFU) enumeration (data are normalized to heat-inactivated control) (*n* = 5). Significance between conditions was identified by multiple *t* tests using the Holm–Sidak method (α = 0.05) to correct for multiple comparisons (****, *p* < 0.000001). (**B**) CFU enumeration of biofilms following the same conditions as planktonic bacteria. Significance difference were tested for using two-way analysis of variance (ANOVA) and the Sidak method to correct for multiple comparisons (*n* = 4, daily experiments conducted in quintuplicate). Error bars indicate standard deviation (SD).

**Figure 2:**
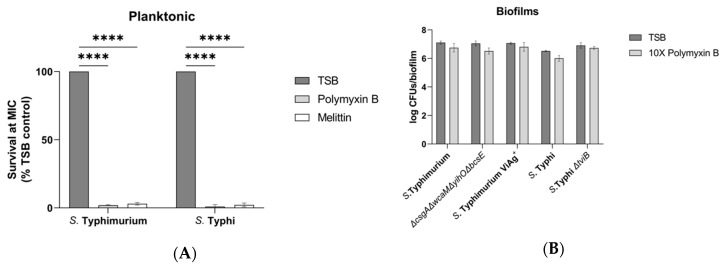

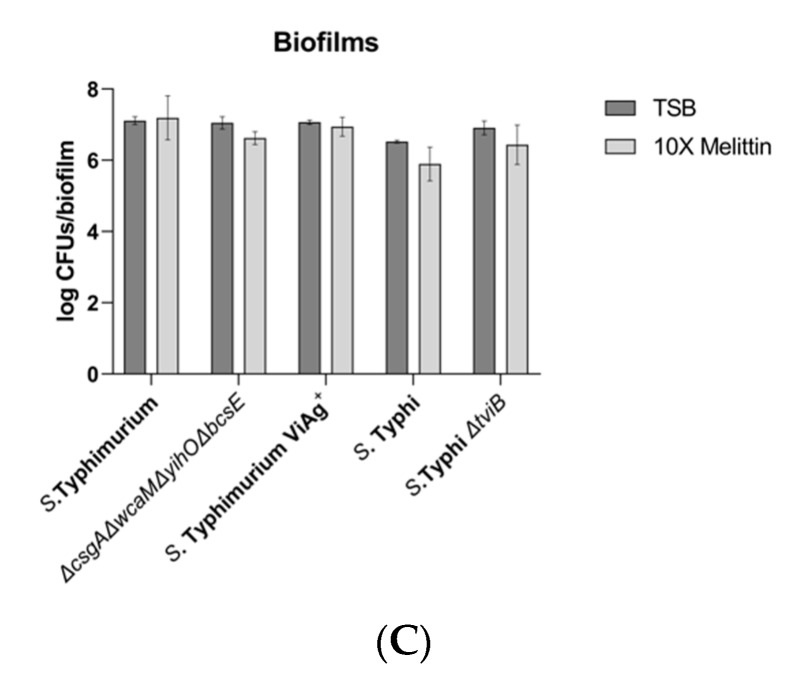
Sensitivity and tolerance to antimicrobial peptides (AMPs). JSG698 used for WT *S*. Typhi. (**A**) Growth from input of WT planktonic *Salmonella* after 2-hour exposure to each AMP at the minimum inhibitory concentration (MIC) determined by CFU enumeration (data are normalized to AMP-free control) (*n* = 3). *S*. Typhimurium challenged with 0.49 µg/mL polymyxin B and 10 µg/mL melittin. *S*. Typhi challenged with 0.24 µg/mL polymyxin B and 10 µg/mL melittin. Significance calculated by two-way ANOVA with Dunnett’s multiple comparison test (***, *p* <0.0005; ****, *p* < 0.0001). (**B**,**C**) Biofilm tolerance to AMPs supplied at 10× the WT planktonic MIC. Significance was tested for by two-way ANOVA with a Sidak multiple comparison correction (*n* = 3, daily experiments conducted in triplicate). Error bars indicate SD.

Cytolytic activity of the complement system membrane attack complex and of AMPs is dependent on direct cell contact. Therefore, these soluble innate immune factors readily target planktonic bacterial surfaces (Figure 1A, Figure 2A) but could be functionally inhibited by EPSs. To address the hypothesis that EPSs are responsible for biofilm tolerance to soluble innate immune factors that successfully target planktonic bacteria, WT and EPS-deficient *S.* Typhimurium (*ΔcsgAΔwcaMΔyihOΔbcsE*) and *S.* Typhi biofilms were challenged with normal human serum and AMPs with the expectation that viable CFUs in EPS-deficient biofilms would be reduced compared to WT biofilms.

WT planktonic *Salmonella* were significantly inhibited by 30% serum, with *S*. Typhimurium 50% inhibited and *S*. Typhi inhibited by 39% (Figure 1A). The same concentration of serum was unable to significantly reduce the viability of WT *Salmonella* biofilm-associated bacteria after 3 hours, even when the four major EPSs (*S.* Typhimurium) or the Vi antigen (*S.* Typhi) had been genetically eliminated (Figure 1B). Further analysis of individual EPS mutants was not conducted because their combined elimination did not produce an altered phenotype. The addition of Vi antigen to *S*. Typhimurium also did not significantly affect serum resistance (Figure 1B). These data suggest that *S*. Typhimurium maintains intrinsic tolerance to complement due to the biofilm lifestyle by unidentified EPSs or other multicellular behaviors.

To test the activity of AMPs, the WT planktonic MIC was experimentally determined by assaying for growth inhibition. For *S.* Typhimurium, the MIC for polymyxin B and melittin was 0.49 µg/ml and 10 µg/ml (respectively). *S.* Typhi had the same melittin MIC but was twice as susceptible to polymyxin B with an MIC of 0.24 µg/ml (Figure 2A). Similar to the serum sensitivity results, the viability of biofilm-associated bacteria was not reduced by challenge with polymyxin B or melittin supplied at 10× the WT planktonic MIC (Figure 2B,C). This result was consistent for WTs from both serovar and for each of the EPS mutants (*S.* Typhimurium *ΔcsgAΔwcaMΔyihOΔbcsE*, *S.* Typhimurium Vi antigen^+^, and *S.* Typhi *ΔtviB*), so further analysis of individual EPS mutants was not conducted. Once again, these data indicate that there may be additional EPSs or biofilm behaviors responsible tolerance to soluble immune components.

#### 3.1.2. Laboratory *S.* Typhi is Representative of Clinical Isolates from Both Acute and Chronic Patients

The majority of EPS mutants were generated and tested in the *S.* Typhimurium serovar because *S.* Typhimurium generates a chronic typhoid-like disease in mice similar to *S.* Typhi in humans and allows for in vivo modeling of a human-restricted pathogen. Since biofilm tolerance data (Figure 1 and Figure 2) indicate that tolerance mechanisms exist beyond the EPS components that were tested, a panel of *S.* Typhi clinical isolates acquired from acute and chronic patients were surveyed to know if the laboratory strain of *S.* Typhi under scrutiny is representative of acute versus chronic patient isolates. When cultured under biofilm-inducing conditions and challenged with 10× polymyxin B, biofilm-associated CFUs were not significantly reduced for any of the 8 acute or 8 chronic isolates (supplemental data; Figure S1A). Similar results were obtained by challenge with 10× melittin, although one acute isolate (Ch-1) did exhibit reduced biofilm-associated CFUs (supplemental data; Figure S1B). Interestingly, when the Vi antigen was eliminated from this isolate, no inhibition from polymyxin B or melittin was observed (supplemental data; Figure S1A,B). Additionally, no major distinctions were evident between the acute patient isolates, the chronic patient isolates, and the laboratory *S.* Typhi WT.

### 3.2. The Innate Immune Response to Salmonella Biofilms

After determining that the four major *Salmonella* EPSs do not play a role in resisting attack by the tested innate immune factors, the possibility that biofilms somehow regulate host immunity was investigated. Given the prominent published anti-inflammatory role of the Vi antigen for planktonic cells [48] compared to the relative lack of phenotypes detected for the other major EPSs in vitro, the Vi antigen was predicted to have a significant role in biofilm inhibition of innate phagocytic cell function.

Conducting these experiments required the collection and normalization of biofilm aggregate populations of similar physical characteristics. The system developed to achieve this goal (scraped biofilms from 96-well plates) was validated by flow cytometry quantifying the size and granularity distribution of the normalized aggregates. While the biofilm aggregates were distinct from planktonic controls, among the biofilm aggregates, no major differences were observed between either serovar or EPS mutants in terms of predominant size, distribution of sizes, or granularity (supplemental data; Figure S2). Using aggregate populations of consistent size and granularity limits the possibility that subsequent experimental outcomes are due to differences in physical interactions between the aggregates and host cells.

#### 3.2.1. Vi Antigen Has a Direct Effect on Macrophage Nitric Oxide Production

**Figure 3:**
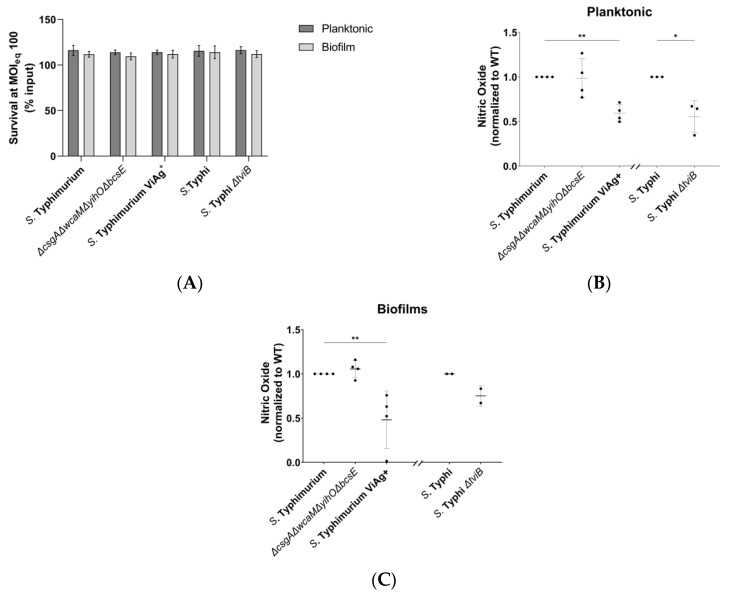
Effect of planktonic and biofilm-associated *Salmonella* on macrophage nitric oxide (NO) production. JSG4383 used for WT *S.* Typhi. (**A**) Planktonic and biofilm aggregate survival determined by CFU enumeration after 2-hour incubation with THP-1 macrophages. Analysis by two-way ANOVA with Sidak correction for multiple comparisons demonstrated no difference in CFU viability between growth states (*n* = 4, daily enumeration conducted in triplicate). (**B**,**C**) NO production by THP-1 macrophages after 2-hour infection with planktonic or biofilm-aggregate *Salmonella.* Significance for *S.* Typhimurium experiments was determined by ordinary one-way ANOVA with Dunnett correction for multiple comparisons (**, *p* < 0.005). *S.* Typhi significant differences were identified by unpaired *t* test (*, *p* < 0.05) (*n* = 4, daily experiments conducted in duplicate). Error bars indicate SD.

Dendritic cells and macrophages are responsible for the delivery of *S.* Typhi from the gut to the liver, and tissue-resident macrophages are presumably the first innate immune cell to interact with *S.* Typhi after it arrives at the gallbladder [21,22,49,50]. Due to these extensive and early interactions between *S.* Typhi and macrophages, *S.* Typhi would be predicted to benefit from the ability to inhibit NO production in vivo. Both planktonic and biofilm infections of macrophages induced peak NO production 2 hours post infection. At these early time points, extracellular planktonic bacteria and biofilm aggregates resisted killing by macrophages equally (Figure 3A). While the four major EPSs did not influence NO production, addition of the Vi antigen to *S.* Typhimurium planktonic and biofilm cultures significantly reduced macrophage NO (Figure 3B,C). Interestingly, elimination of the Vi antigen from both planktonic and biofilm *S.* Typhi cultures showed a similar reduction of NO as was observed with *S.* Typhimurium, instead of the expected increase.

#### 3.2.2. Vi Antigen Inhibition of Neutrophil ROS is Dependent on the Growth State

**Figure 4:**
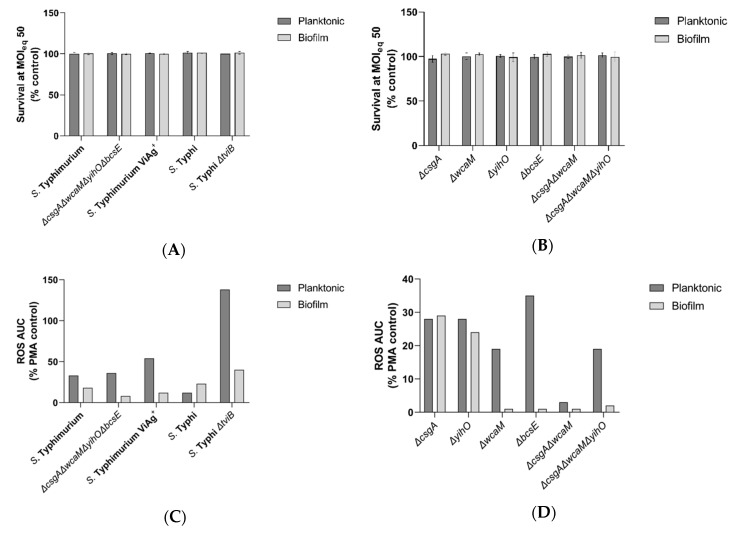
Effect of planktonic and biofilm-associated *Salmonella* on neutrophil ROS production. JSG4383 used for WT *S.* Typhi. (**A**,**B**) CFU enumeration of total (intracellular and extracellular) planktonic and biofilm aggregate survival for each WT and all mutants 80 minutes post-infection. No significant differences in survival were identified by two-way ANOVA with Sidak’s method for multiple comparisons (*n* = 3, enumerated in triplicate). Error bars indicate SD. (**C**,**D**) Reactive oxygen species (ROS) production from neutrophils challenged with planktonic or biofilm aggregates of WT and all EPS mutants. Daily experiments were normalized by a phorbol 12-myristate 13-acetate (PMA)-stimulated control and the data shown are representative of three independent experiments, each with similar trends.

Upon infiltration, neutrophils have the potential to inflict extensive damage through ROS production and other antimicrobial activities. Preventing these functions would enhance biofilm survival in the gallbladder. To determine if EPSs altered neutrophil ROS production, neutrophils were challenged with planktonic bacteria and biofilm aggregates of WT *Salmonella* and EPS-deficient mutants. Similar to the experiments in macrophages, planktonic bacteria and biofilms resisted early killing by neutrophils (Figure 4A,B) with the total CFUs (bacteria inside and outside neutrophils) not different from control conditions, yet the ROS response from neutrophils varied markedly by the microbial growth state (biofilm or planktonic) and EPS mutation (Figure 4C,D). Consistent with studies of planktonic *Salmonella* indicating anti-inflammatory functions of Vi antigen [23,27,29,48,51,52,53], less ROS was produced in response to planktonic *S.* Typhi (Vi antigen present) compared to planktonic *S.* Typhimurium (Vi antigen absent). However, for biofilm aggregates, ROS levels were similar between neutrophils infected with *S.* Typhi and *S.* Typhimurium. As expected, loss of the Vi antigen in both planktonic and biofilm *S.* Typhi resulted in a ROS increase upon infection, although the increase was much higher in planktonic than biofilm infections (Figure 4C). For *S.* Typhimurium producing the Vi antigen (versus *S.* Typhimurium), a decrease in ROS production was expected, but it was only observed with biofilm aggregates and not planktonic bacteria. Thus in most, but not all conditions, Vi antigen suppressed ROS production.

#### 3.2.3. Slime Polysaccharides Have a Role in ROS Stimulation

Surprisingly, a precipitous drop in ROS production was observed with the *S.* Typhimurium *ΔcsgAΔwcaMΔyihOΔbcsE* strain in the biofilm state (Figure 4C). These data indicated a need to investigate individual EPSs that may be responsible for this observation. Loss of curli fimbriae or the O antigen capsule from biofilm aggregates of *S.* Typhimurium eliminated this phenotype and resulted in a ROS response equivalent to infections with their planktonic counterparts (Figure 4D), thus implicating these two EPS components, along with Vi antigen, in the regulation of ROS production in neutrophils.

#### 3.2.4. Biofilms Are Tolerant to H_2_O_2_ but Provide no Protection to ClO^−^

Having identified the Vi antigen as a crucial regulatory component used by *S.* Typhi during both planktonic and biofilm infections, we investigated if mechanisms other than the reported blocking of complement-fixing antibodies [53] might be in play, hypothesizing that the capsule enhances bacterial detoxification pathways and/or inhibits host toxicity pathways. Briefly, host neutrophils produce superoxide through the NADPH oxidase + cytochrome B complex. Bacterial superoxide dismutase converts superoxide to H_2_O_2_, which can be further detoxified to water by catalase or converted to ClO^−^ by myeloperoxidase. ClO^−^ is a precursor for singlet oxygen, peroxynitrites, and chloramines, all of which are potent antimicrobial compounds. By supplying H_2_O_2_ or ClO^−^ independent of host and bacterial functions, the following experiments aimed to identify if one of these divergent pathways in the ROS antimicrobial response was mitigated by *Salmonella*.

**Figure 5:**
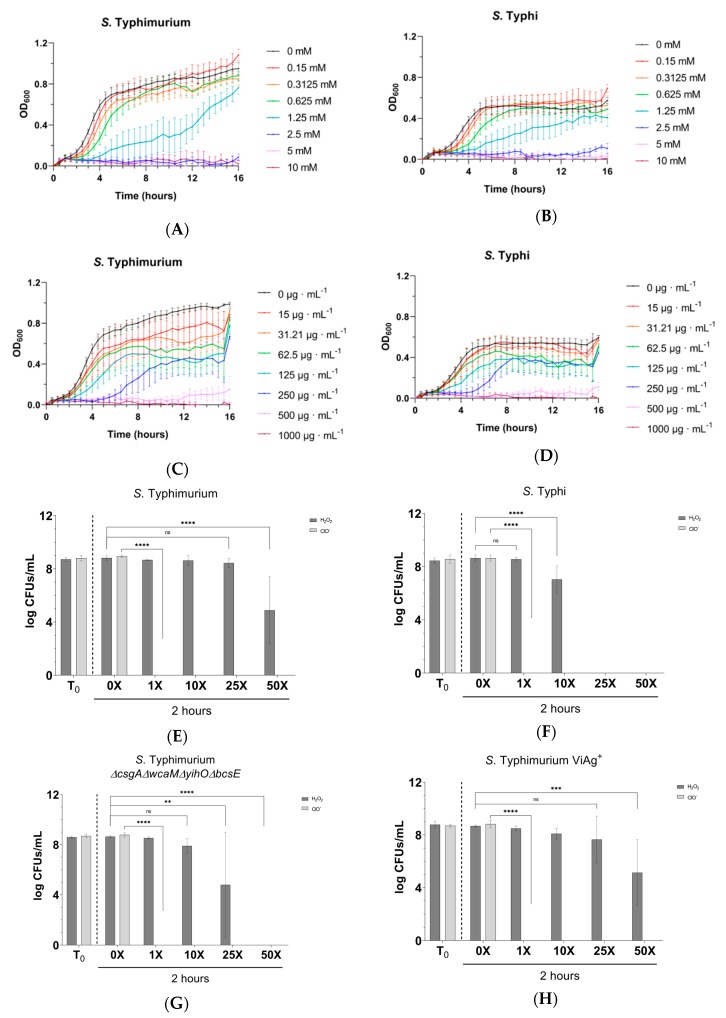

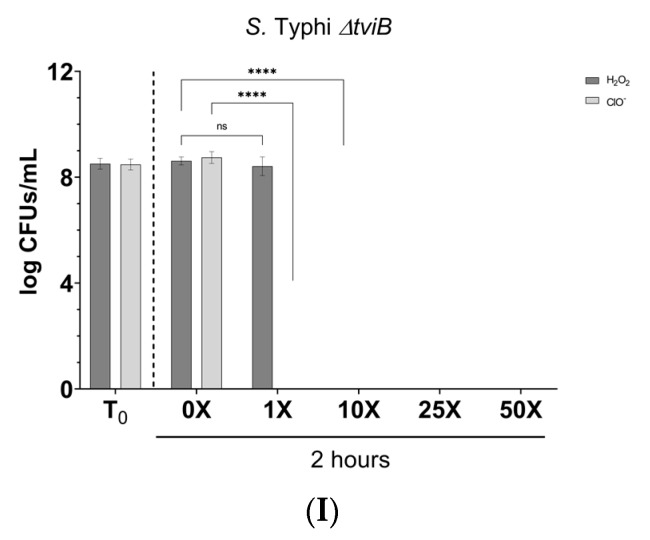
Sensitivity and tolerance to oxidative species. JSG4383 used for WT *S.* Typhi. (**A**,**B**) Growth of WT planktonic *Salmonella* in the presence of H_2_O_2_ demonstrating a minimum inhibitory concentration (MIC) of 2.5 mM for both serovars. (**C**,**D**) Growth of WT planktonic *Salmonella* in the presence of ClO^−^ demonstrating a MIC of 500 µg/ml for both serovars. (**E**–**I**) CFU enumeration of biofilm aggregates challenged with H_2_O_2_ supplied at 1× (2.5 mM), 10× (25 mM), 25× (62.5 mM), or 50× (125 mM) the WT planktonic MIC or ClO^−^ supplied at 1× (0.50 mg/mL), 10× (5 mg/mL), 25× (12.5 mg/mL), or 50× (25 mg/mL) the WT planktonic MIC. Significant reductions in tolerance were identified by comparing control (0×) CFUs with the CFUs of each concentration tested by two-way ANOVA with Tukey’s multiple correction method (**, *p* < 0.01; ***, *p* < 0.0005; ****, *p* < 0.0001). All experiments were conducted in triplicate and the data are the averages of three independent experiments. Error bars indicate SD.

Initial concentration ranges of H_2_O_2_ and ClO^−^ where *Salmonella* demonstrate stratified sensitivity were determined using planktonic bacteria. A challenge was conducted with 5–10 mM H_2_O_2_ or 250–1000 µg/mL ClO^−^ for 60 minutes to determine the best experimental range (supplemental data; Figure S3A,B). Using this data in new experiments, WT planktonic bacteria from both serovars were found to have the same MICs for H_2_O_2_ and ClO^−^: 2.5 mM and 500 µg/mL, respectively (Figure 5A–D). Interestingly, the planktonic MIC to H_2_O_2_ was reduced in *S.* Typhi *ΔtviB* (1.25 mM) and enhanced for *S.* Typhimurium Vi antigen^+^ (4.25 mM), but it remained unchanged in *S.* Typhimurium lacking other EPSs (supplemental data; Figures S4 and S5), suggesting the involvement of Vi antigen in planktonic tolerance to H_2_O_2_. The planktonic sensitivity of both serovars to ClO^−^ was unchanged in EPS mutants versus their parental WT, suggesting the involvement of Vi antigen in planktonic tolerance to H_2_O_2._

**Figure 6:**
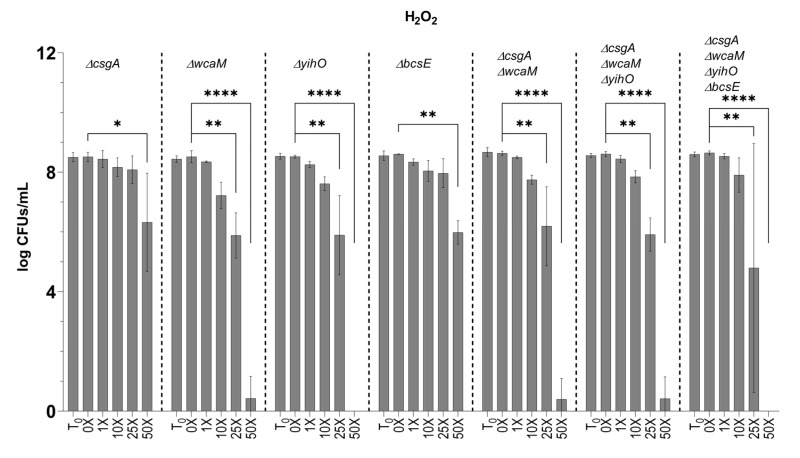
Tolerance of EPS mutants to H_2_O_2_. Biofilm aggregates deficient in one or more EPS(s) were challenged with H_2_O_2_ at the same concentrations detailed above. *S.* Typhimurium *ΔcsgAΔwcaMΔyihOΔbcsE* data were used from Figure 5 for comparative purposes. Significance between CFUs determined by two-way ANOVA with Tukey multiple correction (*, *p* < 0.05; **, *p* <0.01; ****, *p* < 0.0001). Error bars indicate SD.

To determine if EPSs afforded protection to biofilm-resident bacteria, biofilm aggregates were challenged with H_2_O_2_ or ClO^−^ at the WT planktonic MIC or at 10×, 25×, or 50× the MIC concentrations of each compound. Surprisingly, biofilms were as sensitive as planktonic cultures to ClO^−^ as no viable CFUs could be detected beyond challenge at the WT planktonic MIC for either serovar (Figure 5E–I). On the contrary, biofilms of *S.* Typhimurium challenged with H_2_O_2_ at planktonic-lethal doses demonstrated remarkable tolerance. This tolerance was in part due to EPS, as the *S.* Typhimurium *ΔcsgAΔwcaMΔyihOΔbcsE* EPS mutant was more sensitive to H_2_O_2_ (Figure 5E–I). Since a H_2_O_2_ tolerance defect was detected in *S.* Typhimurium *ΔcsgAΔwcaMΔyihOΔbcsE* biofilms (Figure 5G), further analysis of EPS single mutants was conducted (Figure 6) and revealed that the primary EPSs involved in the tolerance phenotype were the O antigen capsule (*ΔyihO*) and colanic acid (*ΔwcaM*). As observed in *S.* Typhimurium, *S.* Typhi biofilms were tolerant to H_2_O_2_ compared to planktonic, although this serovar was notably less tolerant than *S.* Typhimurium (Figure 5E,F,I). Consistent with planktonic data, *S.* Typhi *ΔtviB* biofilms were less tolerant to H_2_O_2_ than WT and survived only at the WT planktonic MIC (Figure 5I).

## 4. Discussion

Our analysis probed for phenotypic differences caused by EPS mutations with two unique perspectives: differences in bacterial survival versus differences in host response. Experiments to determine EPSs that enhance biofilm recalcitrance to selected soluble innate immune factors demonstrated that while the biofilm lifestyle did provide tolerance, an EPS that is solely responsible for inhibiting the bactericidal activities of human serum and AMPs was not identified (Figure 1 and Figure 2). It was hypothesized that EPSs would physically protect biofilm-associated *Salmonella* by preventing diffusion into the biofilm and contact with bacterial membranes, or that they may sequester these molecules through means including electrostatic attraction. However, while EPSs may provide a shield to some innate immune factors, the sensitivity of planktonic cells but robust survival by biofilms deficient for all four major EPSs (*S.* Typhimurium *ΔcsgAΔwcaMΔyihOΔbcsE*, Figure 1 and Figure 2) supports the notion that unknown EPSs or other biofilm-induced surface alterations can result in recalcitrance to humoral immunity.

Dendritic cells and macrophages generate NO via the upregulation of inducible nitric oxide synthase (iNOS) [54,55,56], which is positively stimulated by the pro-inflammatory mediators interleukin-12, interferon-γ, and tumor necrosis factor-α as well as bacterial pathogen-associated molecular patterns [54]. Given the early interaction of dendritic cells and macrophages with planktonic *S.* Typhi and the continued presence of both cell types (planktonic and biofilm) during chronic infection [57], it would be beneficial for the pathogen to limit iNOS activity during the acute and chronic stages of disease. The macrophage NO response to *Salmonella* is independent of curli fimbriae, colanic acid, O antigen capsule, and cellulose (Figure 3B,C). However, we found that the presence of Vi antigen inhibits the NO response to both planktonic and biofilm *Salmonella*, as the addition of Vi antigen to *S.* Typhimurium had an anti-inflammatory effect (Figure 3B,C). Failure by macrophages to exhibit a hyper-inflammatory response to *S.* Typhi *ΔtviB* suggests that *S.* Typhi possesses additional mechanisms for controlling the host response. It is reasonable to expect such a stealthy pathogen to have redundant mechanisms in order to not only control but also fine-tune iNOS activity. The ability to reduce, but not abrogate, iNOS activity would benefit the long-term survival of *S.* Typhi in the gallbladder and fits emerging models for the transition from Th1 to Th2 immunity characteristic of chronic *S.* Typhi infections [57,58,59,60]. Although several studies have demonstrated NO to be a potent and early defense mechanism to many intracellular infections [54,55,56,61,62,63], the global stationary-phase regulator *rpoS* confers *S.* Typhi with a high degree of resistance [55], and a recent study [64] of persistent *Salmonella* found abundant expression of genes for the sensing and detoxification of reactive nitrogen species (RNS) even when the bacteria were in a dormant state. The *S.* Typhi used in our study is *rpoS*^+^ and therefore was expected to be resistant to NO. Furthermore, NO is highly diffusible and has immunosuppressive effects on T and B cell activity [54]. It has long been known that iNOS-deficient macrophages are more likely to become apoptotic when infected by *S.* Typhi [55] and that NO has potent anti-apoptotic activity [55,65,66]. By permitting some NO production, *S.* Typhi may limit the production of RNS to a tolerable concentration while preventing an interferon-driven apoptotic response that would lead to Th1 immunity. While some amount of iNOS activity does lead to an initial Th1 response to gallbladder biofilms, control by *S.* Typhi aids in the development of M2 polarization and the transition to Th2 immunity in the gallbladder.

RAW-264 mouse macrophages treated with the iNOS inhibitor N^ω^-monomethyl L-arginine (L-NMMA) and iNOS^−/^^−^ mice are still able to resist *S.* Typhi infection [55]. In these cases, the antimicrobial activity is mediated by superoxide activity as ROS production occurs independent of iNOS. We chose to examine neutrophil ROS activity because it has been reported that iNOS enhances neutrophil rolling, adhesion, and migration (diapedesis) [67,68], and we have previously found neutrophils in the gallbladder of chronically-infected mice [57]. Although expression of the Vi antigen from planktonic *S.* Typhi has previously been shown to inhibit neutrophil chemotaxis and ROS production [48], it was unknown if this function also occurs in biofilms. Addition of the Vi antigen to planktonic *S.* Typhimurium failed to reduce ROS production from neutrophils, but the presence of Vi antigen in biofilms did cause a decrease in ROS production (Figure 4C). As expected, planktonic *S.* Typhi *ΔtviB* elicited a robust response from neutrophils. Although the response to biofilms from the same mutant was also greater than the response to WT biofilms, the increase in ROS production was surprisingly less dramatic (Figure 4C). These data indicate that while the planktonic inhibition of neutrophil ROS is dependent on the Vi antigen, ROS inhibition by biofilms is dependent on a combination of Vi antigen and other biofilm functions. This biofilm-dependent enhancement demonstrates how neutrophil recruitment may prevent *S.* Typhi from causing systemic disease (which would require the release of planktonic bacteria) but simultaneously is unable to clear biofilm infections. This type of stalemate is in line with many persistent infections that are restricted but not sterilized by the host.

Infections with *S.* Typhimurium EPS mutants indicated that the slime polysaccharides cellulose and colanic acid are required for neutrophil recognition of *S.* Typhimurium biofilms (Figure 4D). Considering that *S.* Typhimurium elicits early neutrophil recruitment and inflammatory disease in humans (to a point of self-limiting infection in the gut) and the fact that *S.* Typhi lacks colanic acid, this discovery highlights one way that *S.* Typhi has undergone host specialization and has an advantage in biofilm recalcitrance to innate immunity. On the other hand, both serovars possess cellulose, which presents an interesting question to investigate further. Since we found cellulose to be immune-stimulating, we predict that the localization of cellulose to the host–biofilm interface would reduce biofilm stealth but the presence of other EPS components in vivo prevents this interaction from occurring.

Consistent with a stalemate infection, our data indicated planktonic and biofilm *Salmonella* resisted killing by oxidative species (Figure 4A,B). To determine the degree to which this phenotype is intrinsic to *Salmonella* versus the biofilm lifestyle, we challenged planktonic and biofilm *Salmonella* with exogenously supplied (chemical) oxidative species. We demonstrated that 500 µg/ml ClO^−^ readily kills planktonic and biofilm-associated *Salmonella* alike (Figure 5C–I), indicating that the presence of EPSs does not afford protection to peroxynitrites, chloramines, or singlet oxygen (downstream ROS molecules produced by ClO^−^). However, the survival of biofilm-associated bacteria in conditions with 50-fold more H_2_O_2_ than the WT planktonic MIC (Figure 5A,B,E–I) indicates that EPSs augment biofilm tolerance to the ROS precursor of ClO^−^. We predict that EPSs slow the diffusion of H_2_O_2_ into the biofilm to a rate that permits the effective detoxification of H_2_O_2_ by catalase enzymes expressed by *Salmonella* [69,70,71]. The H_2_O_2_ tolerance defect detected in *S.* Typhimurium *ΔcsgAΔwcaMΔyihOΔbcsE* biofilms (Figure 5E,G) can be attributed specifically to the O antigen capsule and colanic acid (Figure 6). Interestingly, *S.* Typhi *ΔtviB* biofilms also demonstrated a tolerance defect, as they only survived H_2_O_2_ challenge at the planktonic MIC (Figure 5F,I). Consistently, the MIC of planktonic *S.* Typhi *ΔtviB* was reduced to 1.25 mM H_2_O_2_ (supplemental data; Figure S4A); the growth of planktonic *S.* Typhimurium expressing the Vi antigen was reduced—but not inhibited—by the WT H_2_O_2_ MIC, and complete inhibition required 4.25 mM H_2_O_2_ (1.7-fold the WT MIC) (supplemental data; Figure S5). Together, these data implicate biofilm Vi antigen as well as *S.* Typhimurium O antigen capsule and colanic acid in tolerance to oxidative killing.

In our view, it is no coincidence that *S.* Typhi has dispensed colanic acid; even though the polysaccharide augments *S.* Typhimurium tolerance to H_2_O_2_, the ability of *S.* Typhi to outright prevent or reduce oxidative pathways by eliminating this immunostimulatory EPS pays far greater dividends. Furthermore, the other *S.* Typhimurium EPS that was identified as important for tolerance to H_2_O_2_ (O antigen capsule) is analogous to the Vi antigen in typhoidal serovars in that it drives H_2_O_2_ tolerance. The discovery that each capsule is responsible for H_2_O_2_ tolerance in its respective serovar fits with our other evidence and highlights how *S.* Typhi relies on the Vi antigen to establish and maintain chronic infections. While *S.* Typhimurium biofilms depend on the O antigen capsule to reduce ROS production and for tolerance to H_2_O_2_, the O antigen capsule was unable to reduce NO production in the same manner that the Vi antigen and *S.* Typhi succeed in reducing NO production. Overall, the evolution of the Vi antigen in typhoidal serovars and the ability to fine-tune iNOS activity sets these human-adapted pathogens apart from other *Salmonella,* as the Vi antigen has a prominent role in regulating the host NO and ROS responses to biofilm infections as well as enhancing biofilm recalcitrance during chronic infections that are bound to be exposed to some level of antimicrobial defenses. We have shown how the Vi antigen represents a key EPS of *S.* Typhi biofilms and that it likely elicits modulation of the innate immune system of chronic carriers.

## 5. Conclusions

We have provided strong evidence that *S.* Typhi inhibits innate immunity, but also acknowledge that host functions can ultimately prevail. Adaptive immunity has been documented in chronic carriers in the form of *Salmonella-*specific circulating antibodies and *Salmonella*-specific T cells [61,64,72,73]. Yet the inability to clear infections by innate or adaptive means indicates a true stalemate infection. From the perspective of the infected individual, a chronic stalemate infection is beneficial in the sense that it is preventing systemic disease. Of course, from a public health standpoint, chronic disease is the apex issue and must be mitigated, but in a manner that does not target biofilms so effectively that they rapidly disburse and cause systemic morbidity. By studying the innate immune interactions of *Salmonella* biofilms, we have demonstrated one potential pathway in which innate immunity could potentially be enhanced to help achieve this goal and believe that activating myeloperoxidase activity in vivo in response to Vi antigen would benefit the natural ability of host clearance.

## Figures and Tables

**Table 1:** Strains used in this study. EPS: extracellular polymeric substances.

Strain	Genotype	EPS Deficiency	Reference Source
JSG210	WT *S.* Typhimurium	-	ATCC14028
JSG3736	*ΔcsgA*	Curli fimbriae	[37]
JSG3742	*ΔwcaM*	Colanic acid	[37]
JSG3672	*ΔyihO*	O antigen capsule	[37]
JSG3838	*ΔbcsE*	Cellulose	[37]
JSG3790	*ΔcsgAΔwcaM*	Curli fimbriae, Colanic acid	[37]
JSG3829	*ΔcsgAΔwcaMΔyihO*	Curli fimbriae, Colanic acid, O antigen capsule	[37]
JSG3841	*ΔcsgAΔwcaMΔyihOΔbcsE*	Curli fimbriae, Colanic acid, O antigen capsule, Cellulose	[37]
JSG3738	*S.* Typhimurium + pTH170 (*viaB*)	Vi antigen^+^ (ViAg^+^)	[24]
JSG698	WT *S*. Typhi	-	Ty2
JSG4383	WT *S.* Typhi *rpoS^+^*	-	[40]
JSG1213	*S.* Typhi *ΔtviB*	Vi antigen	[41]
JSG4123	*S*. Typhi Ch-1 *ΔtviB*	Vi antigen	This study

**Table 2:** Clinical isolates used in this study.

Strain	Designation	Geographic Source	Isolation Site
JSG3074	Ch-1	Mexico City	Gallstone
JSG3076	Ch-2	Mexico City	Gallbladder tissue
JSG3979	Ch-3	Vietnam	Gallbladder
JSG3980	Ch-4	Vietnam	Gallbladder
JSG3981	Ch-5	Vietnam	Gallbladder
JSG3982	Ch-6	Vietnam	Gallbladder
JSG3983	Ch-7	Vietnam	Gallbladder
JSG3984	Ch-8	Vietnam	Gallbladder
JSG3985	Ac-1	Vietnam	Unspecified
JSG3986	Ac-2	Vietnam	Unspecified
JSG3987	Ac-3	Vietnam	Unspecified
JSG3988	Ac-4	Vietnam	Unspecified
JSG3989	Ac-5	Vietnam	Unspecified
JSG3990	Ac-6	Vietnam	Unspecified
JSG3395	Ac-7	Ohio Department of Health	Blood
JSG3400	Ac-8	Ohio Department of Health	Bile

**Table 3:** Oligonucleotide primers used in this study.

Primer	Sequence	Purpose
JG2934	**5’—**ATAAAATTTTAGTAAAGGATTAATAAGAGT GTTCGGTATAGTGTAGGCTGGAGCTGCTTC**—3’**	Forward *tviB*
JG2935	**5’—**GTCCGTAGTTCTTCGTAAGCCGTCATGATT ACAATCTCACCATATGAATATCCTCCTTAG**—3’**	Reverse *tviB*
JG2936	**5’—**TCAGCGACTTCTGTTCTATTCAAGTAAGAAAGGGGTACGG**—3’**	Forward verification *tviB*
JG2937	**5’—**GCTCCTCACTGACGGACGTGCGAACGTCGTCTAGATTATG**—3’**	Reverse verification *tviB*

## Supplementary Materials

The following are available online at https://www.mdpi.com/2076-2607/8/2/253/s1, Figure S1: Biofilm tolerance of *S.* Typhi clinical isolates, Figure S2: Analysis of biofilm aggregates, Figure S3: Planktonic sensitivity to oxidative species, Figure S4: EPS mutant planktonic MICs, Figure S5: *S*. Typhimurium Vi antigen^+^ planktonic MIC (H_2_O_2_).

## References

[1] Gonzalez-Escobedo G., Marshall J.M., Gunn J.S. (2010). Chronic and acute infection of the gall bladder by *Salmonella* Typhi: understanding the carrier state. Nat. Rev. Microbiol..

[2] Crawford R.W., Reeve K.E., Gunn J.S. (2010). Flagellated but Not Hyperfimbriated *Salmonella enterica* Serovar Typhimurium Attaches to and Forms Biofilms on Cholesterol-Coated Surfaces. J. Bacteriol..

[3] Crawford R.W., Rosales-Reyes R., Ramirez-Aguilar Mde L., Chapa-Azuela O., Alpuche-Aranda C., Gunn J.S. (2010). Gallstones play a significant role in *Salmonella* spp. gallbladder colonization and carriage. Proc. Natl. Acad. Sci. U.S.A..

[4] Gunn J.S., Marshall J.M., Baker S., Dongol S., Charles R.C., Ryan E.T. (2014). *Salmonella* chronic carriage: epidemiology, diagnosis, and gallbladder persistence. Trends Microbiol..

[5] Gunn J.S., Bakaletz L.O., Wozniak D.J. (2016). What’s on the Outside Matters: The Role of the Extracellular Polymeric Substance of Gram-negative Biofilms in Evading Host Immunity and as a Target for Therapeutic Intervention. J. Biol. Chem..

[6] Johnson R., Ravenhall M., Pickard D., Dougan G., Byrne A., Frankel G. (2018). Comparison of *Salmonella enterica* serovars Typhi and Typhimurium reveals typhoidal serovar-specific responses to bile. Infect. Immun..

[7] Hay A., Zhu J. (2016). In Sickness and in Health: The relationships between bacteria and bile in the human gut. Advances in applied microbiology.

[8] González J.F., Tucker L., Fitch J., Wetzel A., White P., Gunn J.S. (2019). Human bile-mediated regulation of *Salmonella* curli fimbriae. J. Bacteriol..

[9] Chin K.C.J., Taylor T.D., Hebrard M., Anbalagan K., Dashti M.G., Phua K.K. (2017). Transcriptomic study of *Salmonella enterica* subspecies *enterica* serovar Typhi biofilm. BMC Genom..

[10] Deksissa T., Gebremedhin E.Z. (2019). A cross-sectional study of enteric fever among febrile patients at Ambo hospital: Prevalence, risk factors, comparison of Widal test and stool culture and antimicrobials susceptibility pattern of isolates. BMC Infect. Dis..

[11] Mawazo A., Bwire G.M., Matee M.I. (2019). Performance of Widal test and stool culture in the diagnosis of typhoid fever among suspected patients in Dar es Salaam, Tanzania. BMC Res. Notes.

[12] Arora P., Thorlund K., Brenner D.R., Andrews J.R. (2019). Comparative accuracy of typhoid diagnostic tools: A Bayesian latent-class network analysis. Plos Negl. Trop. Dis..

[13] Chang M.S., Woo J.H., Kim S. (2019). Management of Typhoid Fever–Clinical and Historical Perspectives in Korea. Infect. Chemother..

[14] González J.F., Alberts H., Lee J., Doolittle L., Gunn J.S. (2018). Biofilm Formation Protects *Salmonella* from the Antibiotic Ciprofloxacin In Vitro and In Vivo in the Mouse Model of chronic Carriage. Sci. Rep..

[15] Stanaway J.D., Reiner R.C., Blacker B.F., Goldberg E.M., Khalil I.A., Troeger C.E., Andrews J.R., Bhutta Z.A., Crump J.A., Im J. (2019). The global burden of typhoid and paratyphoid fevers: a systematic analysis for the Global Burden of Disease Study 2017. Lancet Infect. Dis..

[16] Crump J.A., Luby S.P., Mintz E.D. (2004). The global burden of typhoid fever. Bull. World Health Organ..

[17] Kirk M.D., Pires S.M., Black R.E., Caipo M., Crump J.A., Devleesschauwer B., Döpfer D., Fazil A., Fischer-Walker C.L., Hald T. (2015). World Health Organization estimates of the global and regional disease burden of 22 foodborne bacterial, protozoal, and viral diseases, 2010: a data synthesis. Plos Med..

[18] Bäumler A., Fang F.C. (2013). Host specificity of bacterial pathogens. Cold Spring Harb Perspect. Med..

[19] Wain J., House D., Parkhill J., Parry C., Dougan G. (2002). Unlocking the genome of the human typhoid bacillus. Lancet Infect. Dis..

[20] Langridge G.C., Fookes M., Connor T.R., Feltwell T., Feasey N., Parsons B.N., Seth-Smith H.M., Barquist L., Stedman A., Humphrey T. (2015). Patterns of genome evolution that have accompanied host adaptation in *Salmonella*. Proc. Natl. Acad. Sci..

[21] Kurtz J.R., Goggins J.A., McLachlan J.B. (2017). *Salmonella* infection: Interplay between the bacteria and host immune system. Immunol. Lett..

[22] Hurley D., McCusker M.P., Fanning S., Martins M. (2014). *Salmonella*–host interactions–modulation of the host innate immune system. Front. Immunol..

[23] Raffatellu M., Santos R.L., Chessa D., Wilson R.P., Winter S.E., Rossetti C.A., Lawhon S.D., Chu H., Lau T., Bevins C.L. (2007). The capsule encoding the *viaB* locus reduces interleukin-17 expression and mucosal innate responses in the bovine intestinal mucosa during infection with *Salmonella enterica* serotype Typhi. Infect. Immun..

[24] Haneda T., Winter S.E., Butler B.P., Wilson R.P., Tükel Ç., Winter M.G., Godinez I., Tsolis R.M., Bäumler A.J. (2009). The capsule-encoding *viaB* locus reduces intestinal inflammation by a *Salmonella* pathogenicity island 1-independent mechanism. Infect. Immun..

[25] Winter S.E., Raffatellu M., Wilson R.P., Rüssmann H., Bäumler A.J. (2008). The *Salmonella enterica* serotype Typhi regulator TviA reduces interleukin-8 production in intestinal epithelial cells by repressing flagellin secretion. Cell. Microbiol..

[26] Crawford R.W., Keestra A.M., Winter S.E., Xavier M.N., Tsolis R.M., Tolstikov V., Bäumler A.J. (2012). Very long O-antigen chains enhance fitness during *Salmonella*-induced colitis by increasing bile resistance. Plos Pathog..

[27] Wilson R.P., Winter S.E., Spees A.M., Winter M.G., Nishimori J.H., Sanchez J.F., Nuccio S.P., Crawford R.W., Tukel C., Baumler A.J. (2011). The Vi capsular polysaccharide prevents complement receptor 3-mediated clearance of *Salmonella enterica* serotype Typhi. Infect. Immun..

[28] Tükel Ç., Raffatellu M., Humphries A.D., Wilson R.P., Andrews-Polymenis H.L., Gull T., Figueiredo J.F., Wong M.H., Michelsen K.S., Akçelik M. (2005). CsgA is a pathogen-associated molecular pattern of *Salmonella enterica* serotype Typhimurium that is recognized by Toll-like receptor 2. Mol. Microbiol..

[29] Raffatellu M., Chessa D., Wilson R.P., Dusold R., Rubino S., Baumler A.J. (2005). The Vi capsular antigen of *Salmonella enterica* serotype Typhi reduces Toll-like receptor-dependent interleukin-8 expression in the intestinal mucosa. Infect. Immun..

[30] Parween F., Yadav J., Qadri A. (2019). The virulence polysaccharide of *Salmonella* Typhi suppresses activation of Rho family GTPases to limit inflammatory responses from epithelial cells. Front. Cell. Infect. Microbiol..

[31] Leid J.G. (2009). Bacterial biofilms resist key host defenses. Microbe.

[32] Valentini M., Filloux A. (2016). Biofilms and Cyclic di-GMP (c-di-GMP) Signaling: Lessons from *Pseudomonas aeruginosa* and Other Bacteria. J. Biol. Chem..

[33] Van Acker H., Coenye T. (2016). The Role of Efflux and Physiological Adaptation in Biofilm Tolerance and Resistance. J. Biol. Chem..

[34] Keestra-Gounder A.M., Tsolis R.M., Bäumler A.J. (2015). Now you see me, now you don’t: The interaction of *Salmonella* with innate immune receptors. Nat. Rev. Microbiol..

[35] Pestrak M.J., Chaney S.B., Eggleston H.C., Dellos-Nolan S., Dixit S., Mathew-Steiner S.S., Roy S., Parsek M.R., Sen C.K., Wozniak D.J. (2018). *Pseudomonas aeruginosa* rugose small-colony variants evade host clearance, are hyper-inflammatory, and persist in multiple host environments. Plos Pathog..

[36] Leid J.G., Willson C.J., Shirtliff M.E., Hassett D.J., Parsek M.R., Jeffers A.K. (2005). The exopolysaccharide alginate protects *Pseudomonas aeruginosa* biofilm bacteria from IFN-γ-mediated macrophage killing. J. Immunol..

[37] Adcox H.E., Vasicek E.M., Dwivedi V., Hoang K.V., Turner J., Gunn J.S. (2016). *Salmonella* Extracellular Matrix Components Influence Biofilm Formation and Gallbladder Colonization. Infect. Immun..

[38] Kostakioti M., Hadjifrangiskou M., Hultgren S.J. (2013). Bacterial biofilms: development, dispersal, and therapeutic strategies in the dawn of the postantibiotic era. Cold Spring Harb Perspect.Med..

[39] Coynault C., Robbe-Saule V., Norel F. (1996). Virulence and vaccine potential of *Salmonella* typhimurium mutants deficient in the expression of the RpoS (σs) regulon. Mol. Microbiol..

[40] Santander J., Wanda S.-Y., Nickerson C.A., Curtiss R. (2007). Role of RpoS in fine-tuning the synthesis of Vi capsular polysaccharide in *Salmonella enterica* serotype Typhi. Infect. Immun..

[41] Virlogeux I., Waxin H., Ecobichon C., Popoff M.Y. (1995). Role of the *viaB* locus in synthesis, transport and expression of *Salmonella* typhi Vi antigen. Microbiology.

[42] Datsenko K.A., Wanner B.L. (2000). One-step inactivation of chromosomal genes in *Escherichia coli* K-12 using PCR products. Proc. Natl. Acad. Sci. USA.

[43] Cherepanov P.P., Wackernagel W. (1995). Gene disruption in *Escherichia coli*: TcR and KmR cassettes with the option of Flp-catalyzed excision of the antibiotic-resistance determinant. Gene.

[44] Tucker K.A., Lilly M.B., Heck L., Rado T.A. (1987). Characterization of a new human diploid myeloid leukemia cell line (PLB-985) with granulocytic and monocytic differentiating capacity. Blood.

[45] Drexler H.G., Dirks W.G., Matsuo Y., MacLeod R.A.F. (2003). False leukemia–lymphoma cell lines: an update on over 500 cell lines. Leukemia.

[46] Pedruzzi E., Fay M., Elbim C., Gougerot-Pocidalo M.A. (2002). Differentiation of PLB-985 myeloid cells into mature neutrophils, shown by degranulation of terminally differentiated compartments in response to N-formyl peptide and priming of superoxide anion production by granulocyte–macrophage colony-stimulating factor. Br. J. Haematol..

[47] Pivot-Pajot C., Chouinard F.C., El Azreq M.A., Harbour D., Bourgoin S.G. (2010). Characterisation of degranulation and phagocytic capacity of a human neutrophilic cellular model, PLB-985 cells. Immunobiology.

[48] Hiyoshi H., Wangdi T., Lock G., Saechao C., Raffatellu M., Cobb B.A., Bäumler A.J. (2018). Mechanisms to evade the phagocyte respiratory burst arose by convergent evolution in typhoidal *Salmonella* serovars. Cell Rep..

[49] Bravo-Blas A., Utriainen L., Clay S.L., Kästele V., Cerovic V., Cunningham A.F., Henderson I.R., Wall D.M., Milling S.W. (2019). *Salmonella enterica* serovar typhimurium travels to mesenteric lymph nodes both with host cells and autonomously. J. Immunol..

[50] Hume P.J., Singh V., Davidson A.C., Koronakis V. (2017). Swiss army pathogen: The *Salmonella* entry toolkit. Front. Cell. Infect. Microbiol..

[51] Jansen A.M., Hall L.J., Clare S., Goulding D., Holt K.E., Grant A.J., Mastroeni P., Dougan G., Kingsley R.A. (2011). A *Salmonella* Typhimurium-Typhi genomic chimera: A model to study Vi polysaccharide capsule function in vivo. Plos Pathog..

[52] Sharma A., Qadri A. (2004). Vi polysaccharide of *Salmonella* typhi targets the prohibitin family of molecules in intestinal epithelial cells and suppresses early inflammatory responses. Proc. Natl. Acad. Sci..

[53] Wangdi T., Lee C.-Y., Spees A.M., Yu C., Kingsbury D.D., Winter S.E., Hastey C.J., Wilson R.P., Heinrich V., Bäumler A.J. (2014). The Vi capsular polysaccharide enables *Salmonella enterica* serovar Typhi to evade microbe-guided neutrophil chemotaxis. Plos Pathog..

[54] BIEDZKA-SAREK M., El Skurnik M. (2006). How to outwit the enemy: dendritic cells face *Salmonella*. Apmis.

[55] Alam M.S., Zaki M.H., Yoshitake J., Akuta T., Ezaki T., Akaike T. (2006). Involvement of *Salmonella enterica* serovar Typhi RpoS in resistance to NO-mediated host defense against serovar Typhi infection. Microb. Pathog..

[56] Jiang P., Yang W., Jin Y., Huang H., Shi C., Jiang Y., Wang J., Kang Y., Wang C., Yang G. (2019). *Lactobacillus reuteri* protects mice against *Salmonella* typhimurium challenge by activating macrophages to produce nitric oxide. Microb. Pathog..

[57] González J.F., Kurtz J., Bauer D.L., Hitt R., Fitch J., Wetzel A., La Perle K., White P., McLachlan J., Gunn J.S. (2019). Establishment of Chronic Typhoid Infection in a Mouse Carriage Model Involves a Type 2 Immune Shift and T and B Cell Recruitment to the Gallbladder. MBio.

[58] Muraille E., Leo O., Moser M. (2014). TH1/TH2 paradigm extended: macrophage polarization as an unappreciated pathogen-driven escape mechanism?. Front. Immunol..

[59] Eisele N.A., Ruby T., Jacobson A., Manzanillo P.S., Cox J.S., Lam L., Mukundan L., Chawla A., Monack D.M. (2013). *Salmonella* require the fatty acid regulator PPARδ for the establishment of a metabolic environment essential for long-term persistence. Cell Host Microbe.

[60] McCoy M.W., Moreland S.M., Detweiler C.S. (2012). Hemophagocytic macrophages in murine typhoid fever have an anti-inflammatory phenotype. Infect. Immun..

[61] Monack D.M., Bouley D.M., Falkow S. (2004). *Salmonella* typhimurium persists within macrophages in the mesenteric lymph nodes of chronically infected Nramp1+/+ mice and can be reactivated by IFNγ neutralization. J. Exp. Med..

[62] Frawley E.R., Karlinsey J.E., Singhal A., Libby S.J., Doulias P.-T., Ischiropoulos H., Fang F.C. (2018). Nitric oxide disrupts zinc homeostasis in *Salmonella enterica* serovar Typhimurium. MBio.

[63] Khan S., Fujii S., Matsunaga T., Nishimura A., Ono K., Ida T., Ahmed K.A., Okamoto T., Tsutsuki H., Sawa T. (2018). Reactive persulfides from *Salmonella* Typhimurium downregulate autophagy-mediated innate immunity in macrophages by inhibiting electrophilic signaling. Cell Chem. Biol..

[64] Goldberg M.F., Roeske E.K., Ward L.N., Pengo T., Dileepan T., Kotov D.I., Jenkins M.K. (2018). *Salmonella* Persist in Activated Macrophages in T Cell-Sparse Granulomas but Are Contained by Surrounding CXCR3 Ligand-Positioned Th1 Cells. Immunity.

[65] Alam M.S., Akaike T., Okamoto S., Kubota T., Yoshitake J., Sawa T., Miyamoto Y., Tamura F., Maeda H. (2002). Role of nitric oxide in host defense in murine salmonellosis as a function of its antibacterial and antiapoptotic activities. Infect. Immun..

[66] Akaike T. (2001). Role of free radicals in viral pathogenesis and mutation. Rev. Med Virol..

[67] Nolan S., Dixon R., Norman K., Hellewell P., Ridger V. (2008). Nitric oxide regulates neutrophil migration through microparticle formation. Am. J. Pathol..

[68] Hossain M., Qadri S.M., Liu L. (2012). Inhibition of nitric oxide synthesis enhances leukocyte rolling and adhesion in human microvasculature. J. Inflamm..

[69] Walawalkar Y.D., Vaidya Y., Nayak V. (2016). Response of *Salmonella* Typhi to bile-generated oxidative stress: implication of quorum sensing and persister cell populations. Pathog. Dis..

[70] Tsolis R.M., Bäumler A.J., Heffron F. (1995). Role of *Salmonella* typhimurium Mn-superoxide dismutase (SodA) in protection against early killing by J774 macrophages. Infect. Immun..

[71] Farr S.B., Kogoma T. (1991). Oxidative stress responses in *Escherichia coli* and *Salmonella* typhimurium. Microbiol. Mol. Biol. Rev..

[72] Ravindran R., Foley J., Stoklasek T., Glimcher L.H., McSorley S.J. (2005). Expression of T-bet by CD4 T cells is essential for resistance to *Salmonella* infection. J. Immunol..

[73] Nauciel C., Espinasse-Maes F. (1992). Role of gamma interferon and tumor necrosis factor alpha in resistance to *Salmonella* typhimurium infection. Infect. Immun..

