# Supplementary material for: Salmonella Extracellular Polymeric Substances Modulate Innate Phagocyte Activity and Enhance Tolerance of Biofilm-Associated Bacteria to Oxidative Stress
Source: Microorganisms. 2020 Feb 13;8(2):253. doi: 10.3390/microorganisms8020253 (PMC7074811; doi:10.3390/microorganisms8020253)
Supplement: Supplementary file 1 [file microorganisms-08-00253-s001.pdf]

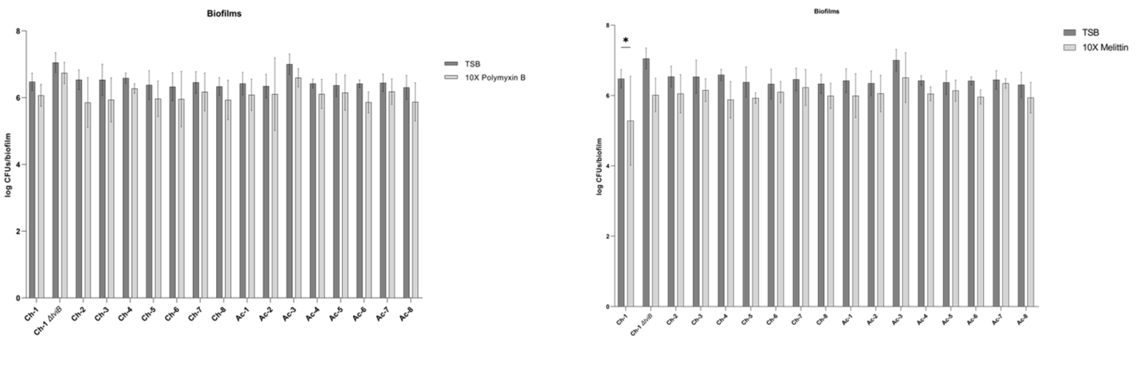

(**A**) (**B**)

Figure 1. Biofilm tolerance of *S.* Typhi clinical isolates. A panel of 8 chronic (Ch-1 to Ch-8) and 8 acute (Ac-1 to Ac-8) clinical isolates challenged with (**A**) polymyxin B or (**B**) melittin supplied at 2.4 µg/ml polymyxin B and 100 µg/mL melittin. Significance was identified by two-way ANOVA with Sidak multiple comparison correction (*, $P < 0.05$) ($n = 3$, daily experiments conducted in triplicate). Error bars indicate SD.

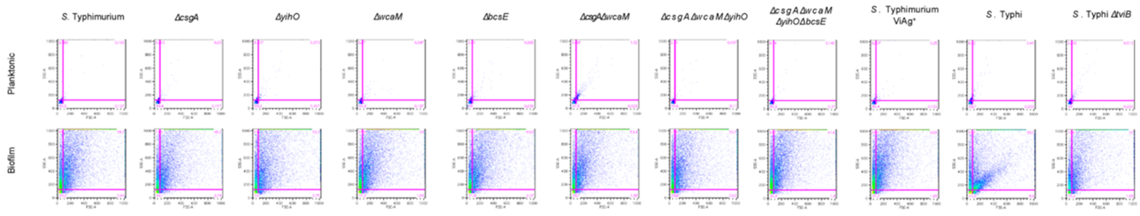

(**A**)

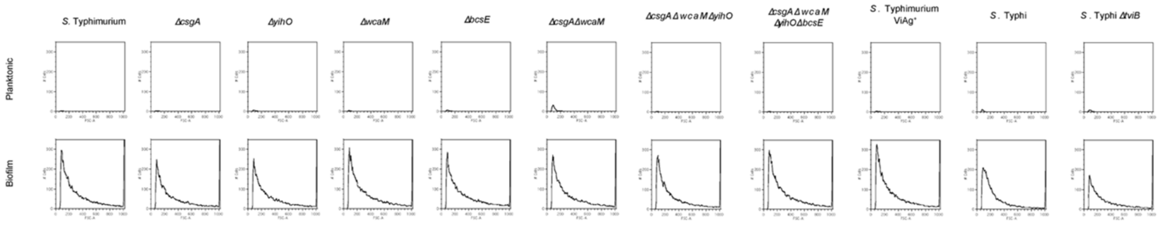

(**B**)

Figure S2. Analysis of biofilm aggregates. Biofilm collection methods yield aggregate populations of consistent size and granularity (**A**). Forward scatter (FSC) and side scatter (SSC) gates were set with planktonic bacteria for each WT and EPS mutant. As expected, the only population detected was small and agranular indicating planktonic bacteria. Applying the gates to biofilm aggregates demonstrates aggregates are distinct from planktonic cells in size and granularity. (**B**) Histograms include events only from quadrant II and quantify the distribution of biofilm aggregate sizes. Aggregates from both serovars and each EPS mutant had similar distribution; small aggregates were predominantly produced but there is a normal distribution as size increases for all strains and thus variation between WTs and mutants is minimal.

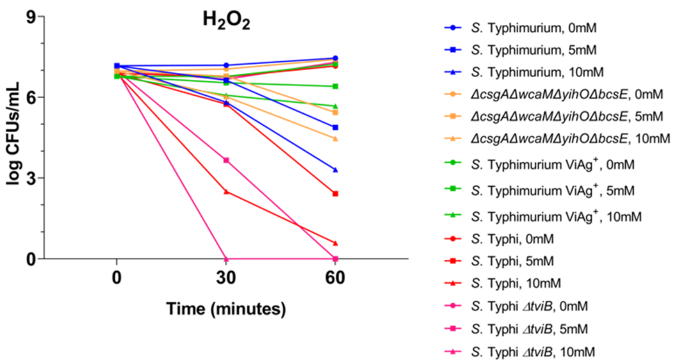

(**A**)

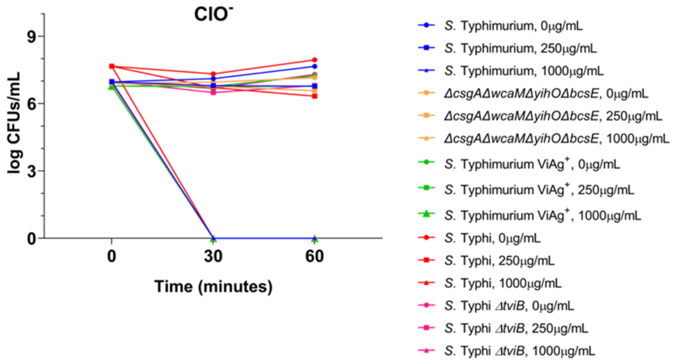

(**B**)

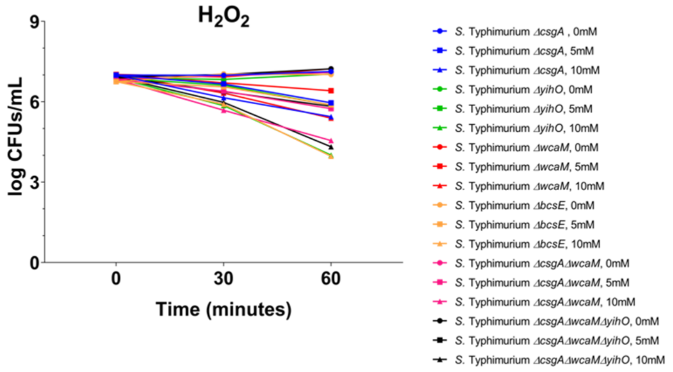

(**C**)

Figure S3. Planktonic sensitivity to oxidative species. JSG4383 used for WT *S.* Typhi. (**A**,**B**) Initial sensitivity tests of planktonic *Salmonella* to H_2O_2 and ClO^- and basis for range of concentrations tested to identify the MIC for each compound. (**C**) Planktonic sensitivity to H_2O_2 for EPS mutants. Each *S.* Typhimurium EPS mutant demonstrated planktonic sensitivity to the same range of H_2O_2 as WT.

H_2O_2

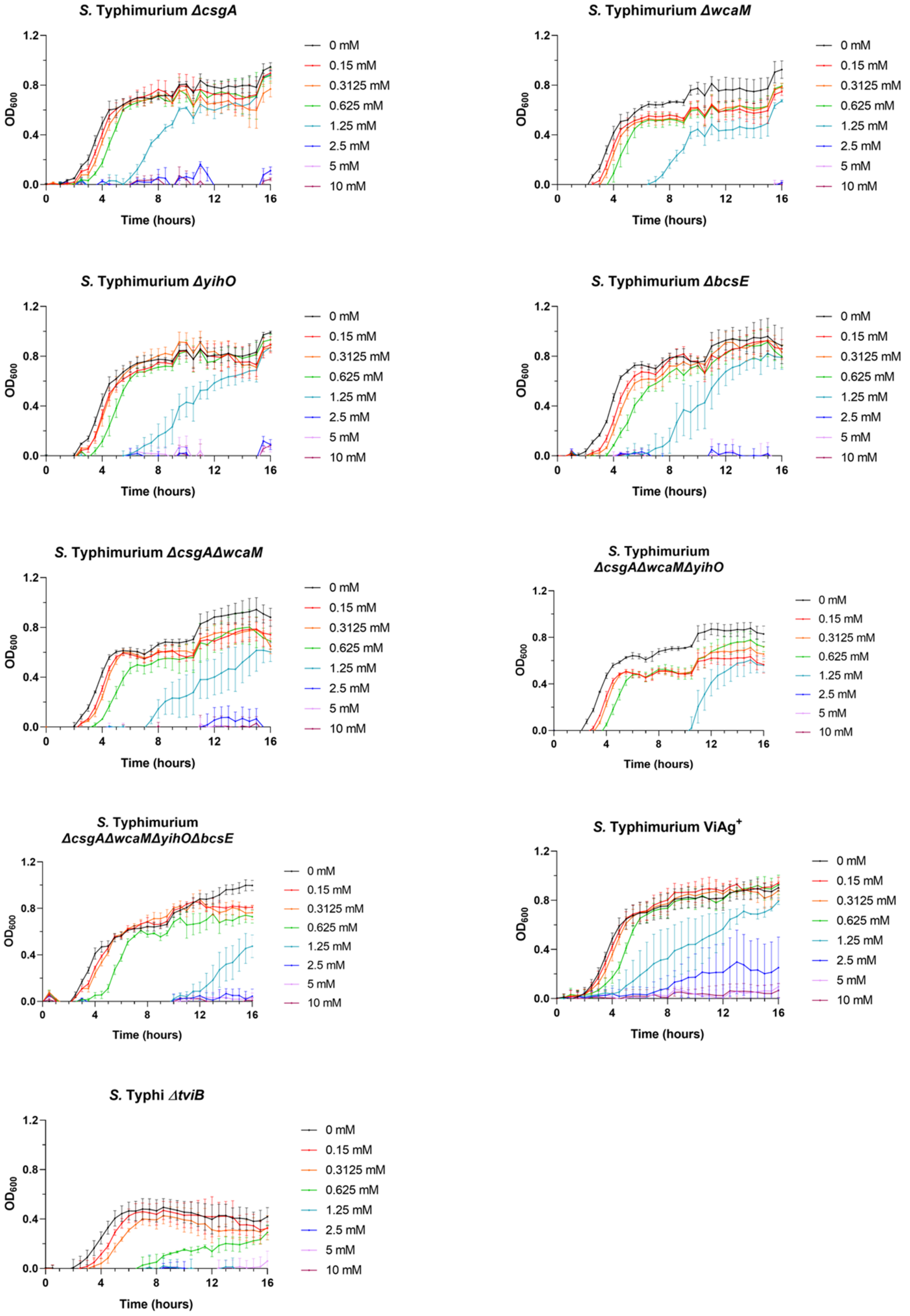
S. Typhimurium ΔcsgA
S. Typhimurium ΔwcaM
S. Typhimurium ΔyihO
S. Typhimurium ΔbcsE
S. Typhimurium ΔcsgAΔwcaM
S. Typhimurium ΔcsgAΔwcaMΔyihO
S. Typhimurium ΔcsgAΔwcaMΔyihOΔbcsE
S. Typhimurium ViAg+
S. Typhi ΔtviB
OD600
Time (hours)
0 mM
0.15 mM
0.3125 mM
0.625 mM
1.25 mM
2.5 mM
5 mM
10 mM

(**A**)

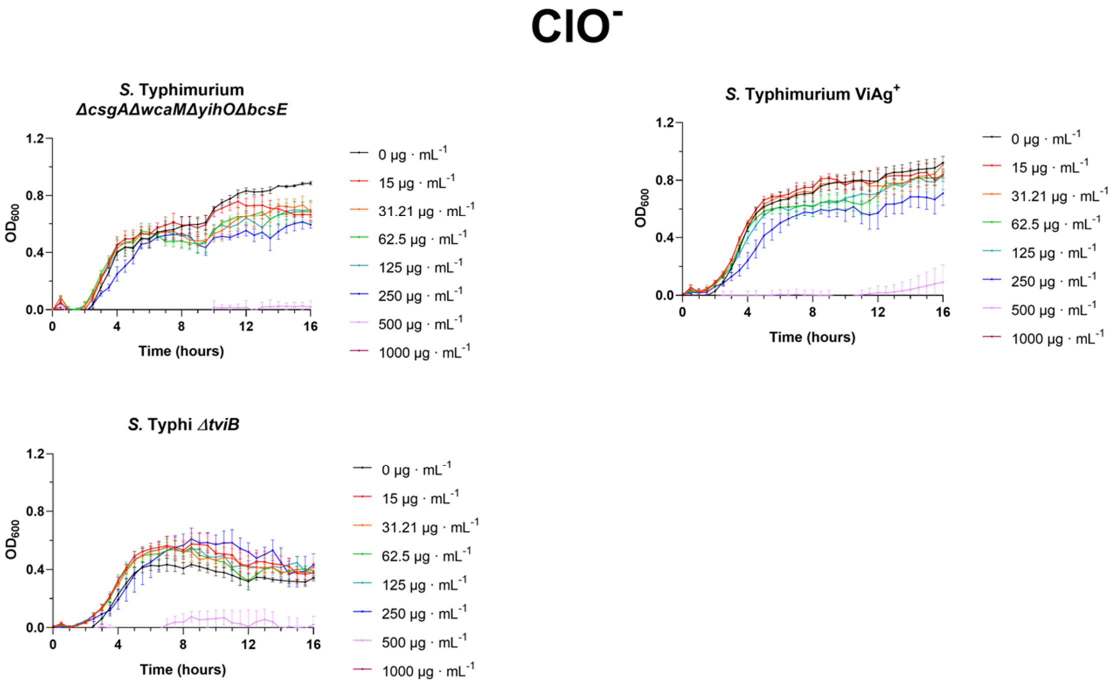

(**B**)

Figure S4. EPS mutant planktonic MICs. The MICs of H_2O_2 and ClO^- was verified for each EPS mutant during planktonic growth. (**A**) The H_2O_2 MIC was equal to WT for *S.* Typhimurium EPS mutants and increased only by the addition of Vi antigen to *S.* Typhimurium. *S.* Typhi *ΔtviB* MIC of H_2O_2 was reduced to half of the WT MIC. (**B**) The ClO^- MIC was equal to WT for each of the EPS mutants tested. Individual *S.* Typhimurium EPS mutants were not investigated since *S.* Typhimurium *ΔcsgAΔwcaMΔyihOΔbcsE* demonstrated no difference from WT.

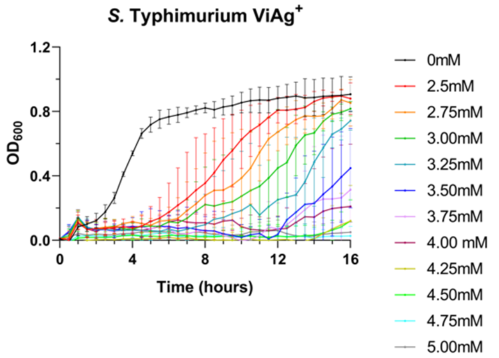

Figure S5. *S.* Typhimurium Vi antigen+ planktonic MIC (H_2O_2). The MIC of H_2O_2 was further investigated for *S.* Typhimurium Vi antigen+ during planktonic growth because it demonstrated growth at the WT MIC (2.5 mM). The exact MIC of *S.* Typhimurium Vi antigen+ was 4.25 mM H_2O_2, indicating a 70% increase in the planktonic MIC.
